# The Synergistic Effects of APOE Genotype and Obesity on Alzheimer’s Disease Risk

**DOI:** 10.3390/ijms20010063

**Published:** 2018-12-24

**Authors:** Nahdia S. Jones, G. William Rebeck

**Affiliations:** Department of Neuroscience, Georgetown University, 3970 Reservoir Rd NW, Washington, DC 20007, USA; nsj10@georgetown.edu

**Keywords:** Apolipoprotein E, obesity, Alzheimer’s disease, cognition, metabolism

## Abstract

The *APOE* gene has three common alleles—E2, E3, and E4, with *APOE4* being the strongest genetic risk factor for developing Alzheimer’s Disease (AD). Obesity is a global epidemic and contributes to multiple metabolic problems. Obesity is also a risk factor for cognitive decline. Here, we review the effects of *APOE4* and obesity on cognition and AD development, independently and together. We describe studies that have associated *APOE4* with cognitive deficits and AD, as well as studies that have associated obesity to cognitive deficits and AD. We then describe studies that have examined the effects of obesity and *APOE* genotypes together, with a focus on *APOE4* and high fat diets. Both human studies and rodent models have contributed to understanding the effects of obesity on the different *APOE* genotypes, and we outline possible underlying mechanisms associated with these effects. Data across approaches support a model in which *APOE4* and obesity combine for greater detrimental effects on metabolism and cognition, in ways that are influenced by both age and sex.

## 1. Alzheimer’s Disease

Alzheimer’s Disease (AD) affects approximately 5 million individuals in the United States and will affect 16 million Americans by 2050 [1]. The disease generally begins with memory loss and progresses to many cognitive domains before death. The neuropathological hallmarks of AD are amyloid plaques and neurofibrillary tangles, which are associated with signs of neuroinflammation. AD pathology manifests itself up to 20 years before symptoms develop, with amyloid accumulation being followed by decreased brain glucose metabolism and neurofibrillary tangles before cognitive symptoms [2,3]. These findings indicate that the disease precedes the symptoms, and with the combination of early pathology and metabolic alterations acting as indicators for AD. Multiple genetic and environmental factors increase AD risk. The largest genetic risk factor is the Apolipoprotein E (*APOE*) E4 allele. Among the environmental factors that affect AD risk, obesity has repeatedly been associated with cognitive decline and AD onset. This review will examine multiple studies that have looked at the effects of *APOE4*, obesity, and their combination on risk and progression of AD. 

## 2. APOE4 and AD

The protein apolipoprotein E (APOE) is a 299 amino acid secreted glycoprotein, produced predominantly by astrocytes in the brain and peripherally by the liver [4]. There are three *APOE* alleles, E2, E3, and E4, encoding proteins that differ by a single amino acid at either amino acid 112 or 158. *APOE2* has an allele frequency in the US of 8%; it is associated with increased risk of cardiovascular diseases and decreased risk of AD [4]. *APOE3* is the most common allele with a frequency of 78%; it is defined as the average risk of AD. *APOE4* has an allele frequency of 14%; *APOE4* homozygotes have 15 times increased risk for AD and heterozygous *APOE3/APOE4* individuals have 3 times increased risk of AD [5]. Peripherally, *APOE4* is also associated with increased risk of metabolic syndrome and cardiovascular disease [6]. While *APOE4* is present in less than 25% of the US population, over 50% of AD patients are *APOE4* carriers [7]. 

In the brain, APOE is involved in cholesterol metabolism and lipid homeostasis [4,5,8]. Its main role is to traffic lipids from astrocytes to neurons; APOE4 is less effective at this trafficking due to its association with smaller lipoproteins than its APOE2 or APOE3 counterparts [5]. Peripherally, APOE2 and APOE3 bind to high-density lipoproteins (HDLs) and are responsible for trafficking lipids to the liver to be eliminated; APOE4 is the least efficient at homeostatic maintenance due to its greater affinity for very low-density lipoproteins [4]. In the AD brain, *APOE4* carriers have increased Aβ accumulation, decreased Aβ clearance [5], and increased aggregation of the toxic oligomeric Aβ [5]. *APOE4* carriers also have increased Aβ inflammatory responses, which may also relate to the increased risk of AD [5].

In addition to the amyloid-related pathological hallmarks of AD, clinical hallmarks include reduced brain glucose uptake in FDG-PET scans. Glucose is required for proper neuronal functioning and its reduced metabolism could underlie *APOE4* deficits in cognition, dendritic spine density and blood–brain barrier (BBB) permeability [9,10,11]. Molecularly, effects of *APOE* genotype on brain glucose could be related to its binding to the low-density lipoprotein receptor-related protein 1 (LRP1). LRP1 is associated with regulation of brain glucose receptors and alterations in brain glucose tolerance [12]. LRP1 is significantly decreased in AD brains [12], perhaps contributing to the reduction in glucose uptake seen in fluorodeoxyglucose-positron emission tomography (FDG-PET) scans of AD patients. 

Sex plays a role in the effect of *APOE* genotype on clinical correlates of AD. Female *APOE4* carriers have impairments on the California verbal learning test and on a verbal fluency test when compared to male *APOE4* carriers [13]. Female *APOE4* carriers showed greater declines in performance IQs when compared to APOE4 noncarriers [14]. Furthermore, with mild cognitive impairment (MCI), only the homozygous male *APOE4* carriers have deficits on delayed recall testing while women with only one of two APOE4 alleles exhibit similar performance reductions [15]. *APOE4* women showed significantly smaller hippocampi compared to APOE4 men carrying an APOE4 allele and non-*APOE4* carriers after MCI [15]. These studies place an emphasis on the increased risk of AD experienced by *APOE4* carriers, with women being more affected than men. 

Studies of *APOE* knock-in (KI) mice have complemented the studies in humans. Control *APOE4* mice and showed increases in amyloid beta (Aβ)42 and tau staining and a decrease in VGlut levels compared to *APOE3* mice [16]. *APOE4* KI mice have decreased spine densities and synaptic integrity at several ages compared to *APOE3* KI mice [10,17,18,19]. Similar decreases in spine densities have been seen in the medial entorhinal cortex of *APOE4* mouse brains [20]. Cognitively, *APOE4* mice have poorer performances in cognitive tasks that test memory acquisition and retention, as compared to their *APOE3* counterparts [20]. *APOE4* mice crossed with familial AD-transgenic mice (E4FAD) have increased Aβ and phosphorylated tau [21]. These mice also exhibit decreases in synaptic proteins and deficits in cognitive tasks [21]. 

Overall, APOE4 is associated with an increase in AD risk, and AD pathology in both human and rodents. Furthermore, without disease manifestation, APOE4 carriers exhibit multiple characteristics that are also seen in AD patients, such as decreases in brain glucose uptake, dendritic spine densities and cognition. 

## 3. Obesity and AD

Obesity affects 600 million adults globally and increases the risks of cognitive deficits [22]. Obesity causes a marked change in systemic homeostasis and lipid metabolism. The most well-recorded phenotype of obesity is an increase in subcutaneous adipose tissue. This is followed by an increase in visceral adipose tissue (VAT), which is more noxious than the subcutaneous form. Increases in VAT are associated with increased risk of AD and cognitive impairment [23,24,25]. VAT is also associated with decreased hippocampal volume and performance on a memory task [26], much like the deficits seen in APOE4 carriers. 

Obesity results in multiple metabolic alterations. Obese individuals have increased blood or plasma cholesterol, glucose, and insulin leading to other diseases such as cardiovascular disease, and diabetes [27,28,29], each independently associated with cognitive deficits. In serum, there is an increase in free fatty acids resulting in increases in LDL and triglycerides, and decreases in HDL [19,30]. Obesity also results in an increase in activated macrophages and secretion of proinflammatory cytokines, TNF-α and IL-6, from the adipose tissue [1,29,31]. These inflammatory alterations are found in cases of middle age obesity, but not obesity at a later age [32]. 

Obesity, particularly in midlife, is associated with MCI and AD [24,33,34,35]. Obesity increases deficits in short term memory, with obese individuals recalling fewer words in the word-list learning task as compared to normal weight individuals [36]. Obese individuals also have deficits in executive functioning [37] and an increased rate of brain atrophy [38]. Improvements in cognitive functioning has been associated with weight loss intervention with obese individuals experiencing low grade improvements in attention and executive functioning [39]. Cognitive improvements in overweight individuals also occurs with weight loss intervention [40] indicating the cognitive deficits can be reversed to an extent. Obese adolescents suffering from metabolic alterations have significantly smaller hippocampal volumes [41,42]; older obese individuals also have significantly smaller hippocampal volumes [43,44]. Cellularly, obesity during midlife has been associated with decreased BBB integrity [45], increased amyloid precursor protein (APP) levels, and increased tau phosphorylation [46,47,48]. All these studies underscore the connection of obesity with cognitive deficits and AD pathology. Furthermore, male patients suffering from Type I diabetes exhibit a significant decrease in cerebral glucose uptake in the thalamus after acute hyperglycemia [49], and patients with Type I and Type II diabetes had slightly (10%) lower brain glucose concentrations [50] suggesting a decrease in glucose metabolism similar to as seen in AD.

Epidemiological studies have noted sex differences in both the development of obesity and the risk of AD. Obese women had an increased risk of AD [51] and there is a positive correlation between higher Body Mass Index (BMI) and inflammation in women [52]. Obesity in women is associated with deficits in BBB integrity at an older age [45], although not at premenopausal ages [53,54]. This protection at earlier ages could be attributed to the availability of estrogen that decreases significantly at middle age with menopause or to the distribution of adipose tissue which changes with age. Initially women develop more subcutaneous adipose tissue while men develop more visceral adipose tissue [23], but as women age the adipose accumulation occurs more around visceral tissue than subcutaneous tissue [13]. The combination of changing adipose tissue distribution and decreases in estrogen levels could explain why obesity can more severely affects women. Decreased testosterone in men increases AD risk, but the association between testosterone and obesity does not reflect the conditions described here [45,51,52]. 

These effects of obesity have not only been seen in humans; multiple rodent studies also display an effect of obesity on AD-related outcomes. *Ob/ob*, *db/db* mice and Zucker rats, rodents with mutations in the genes for leptin or the leptin receptor [55,56,57,58], are genetically altered models of obesity and Type II diabetes. The rodents experience metabolic disorders similar to humans such as significant weight gain from hyperphagia, glucose intolerance, and insulin resistance [59,60,61]. They also have increased fasting plasma and glucose levels, high cholesterol levels and experience cognitive deficits associated with weight gain [59,60,61]. These deficits include increased anxiety-like behavior and decreased spatial memory. During the light dark box task, *Ob/ob* mice spend more time in the dark area [60], and *db/db* display similar behavior when placed on the open field and elevated plus maze [61], indicating that these mice have increased anxiety-like behavior. In the Y-maze the *db/db* mice spent less time exploring the novel arm after a 30 min retention interval [61], demonstrating a deficit in spatial recognition. In the Morris water-maze, both the Zucker rats and *db/db* mice displayed impaired spatial memory acquisition and retention [62]. Molecularly, the *db/db* mice have increased inflammation (increased IL-1β, TNF-α, and IL-6 mRNA) and impairments in synaptic plasticity (long term potentiation (LTP) and long terms depression (LTD)) in the hippocampus [61]. The Zucker rats also have LTP and LTD impairments [61], and *ob/ob* mice have increased levels of hyperphosphorylated tau [59]. These three rodent models demonstrate effects of obesity on neuronal dysfunction.

High fat diets (HFD) are also used to generate models of obesity in rodents. C57/BL6, 3XTgAD, and 5xFAD mice each demonstrate weight gains on HFD [63,64,65]. Wild-type (C57/B6N) mice on HFDs have increased fasting insulin and decreased insulin tolerance [40]. Cognitively, they showed deficits in tasks such as the Morris Water Maze with longer latencies to find the platform and longer swim distances [66]. Elderly C57/BL6 on HFD also show deficits in Y-maze spontaneous alternation [39], indicating diet associated spatial memory deficits. WT mice on high fat/high cholesterol diets have deficits in working memory load [46]. Like in humans, when female mice on HFDs experience dietary weight loss interventions there is an improvement in cognition [67]. The mice experience significant reductions in metabolic disturbances and improvements in object recognition and spatial navigation [67] signifying weight gain and metabolic disturbances are responsible for the cognitive deficits. These cognitive deficits could be due to processes such as inflammation or BBB disruption and the diet intervention could reduce inflammation and BBB permeability. HFD mice showed increases in CD45, Iba1, and GFAP staining, TNF-α, IL-1β, and IL-6 [46], indicating an increase in microglia, astrocytes and cytokines. Rats exposed to a HFD had decreases in BBB integrity, particularly the hippocampus when compared to the prefrontal cortex or striatum [68]. AD mouse models also showed effects of HFD on cognition. 5xFAD mice on HFDs have decreased glucose tolerance compared to the 5xFAD control mice [38], and increased deficits on the Morris Water Maze when compared to 5xFAD and WT mice on control diets and WT mice on the HFD [38]. Similarly, older 3xTgAD mice on a HFD were impaired on the Y-maze spontaneous alternation test when compared to 3xTgAD mice on control diets, and younger 3xTgAD mice on HFDs were impaired in the smell recognition test when compared to 3xTgAD mice on control diets [39]. 3xTgAD mice on HFD with deficits in *n*-3:*n*-6 polyunsaturated fatty acids showed significant increases in insoluble brain Aβ40, and Aβ42 [69]. 3xTgAD mice on HFD also had significant increases in microglial activation in the hippocampus [39] and 5xFAD mice on HFD had increases in oxidative stress in the HPC and cortex [38].

Sex differences in rodents on HFDs have also been reported; however, they differ from those seen in humans. Male mice on HFD had higher fasting glucose and insulin levels and increased abdominal adipose tissue, while female mice only exhibited increased abdominal adipose tissue [70]. Both male and female mice on HFDs had increases in hippocampal Aβ [70]. Male and female mice performed similarly on the Y-maze with a decrease in spontaneous alterations in both [70]. HFD male mice also showed decreases in fear conditioning and passive avoidance tasks when compared to normal male mice, indicating cognitive deficits induced by diet, but these findings were not seen in female mice [71]. Furthermore, male mice on HFDs had significantly lower LTP magnitudes [41] when compared to male mice on normal diets; this finding was not seen between female mice on HFDs and normal diets. Similarly, rats on a HFD showed decreased hippocampal neurogenesis in males but not females [72]. Overall, in rodent models, female mice seem to be protected from the metabolic diseases and the accompanying cognitive deficits [71], possibly due to differences in the adipocytes and sex steroid hormones. Female mice have adipocytes with increased insulin sensitivity and indicators of increased glucose metabolism [73]; when male mice are castrated, their adipocytes exhibit these same traits [73]. Female mice also do not undergo strong alterations in sex hormones like humans do. These finding indicate differences in metabolic changes between sexes but no differences in the effect of obesity on cognition. Thus, while mice are helpful models for studying both the metabolic and cognitive effects of HFD, they do not exactly replicate effects seen in humans. 

## 4. Obesity and APOE 

While both *APOE* genotype and obesity increase cognitive deficits and AD risk individually, few studies have investigated their combined effects. The *APOE4* allele increases the risk for cardiovascular disease and development of metabolic syndromes [6], and a two-fold increase in odds for metabolic syndrome has been noted in homozygous *APOE4* carriers [74]. *APOE4* carriers have significantly higher fasting glucose and insulin levels [75] and an increased risk of metabolic syndrome, with a younger age of onset [74]. These findings were seen without a significant increase in BMI. Rather, *APOE4* carriers have a lower average BMI than *APOE3* or *APOE2* carriers with *APOE2* carriers having the highest BMI (E2 > E3 > E4) [76,77]. Although *APOE2* carriers have the highest BMI, they are protected against AD [78], perhaps due their reduced risk of metabolic syndrome and decreased baseline metabolic perturbances [79,80]. *APOE4* carriers have increased total cholesterol and low-density lipoproteins as compared to other *APOE* genotypes [81,82]. Obese *APOE4* carriers have elevated levels of plasma cholesterol levels, circulating triglycerides, and insulin resistance [83] and obese men with *APOE4* had elevated levels of insulin and glucose. These finding were not seen in non-obese *APOE4* carriers or in individuals with other *APOE* genotypes. Nor was a difference found in women, indicating a potential difference in how sex affects the interaction between obesity and the *APOE4* genotype. These studies show that *APOE2* decreases the risk of metabolic syndrome but not higher BMI, while *APOE4* increases the risk of metabolic syndrome. 

The combination of obesity and *APOE4* is not only associated with negative metabolic effects, but also with negative cognitive effects. Midlife obesity was significantly associated with increased risk of late onset AD in *APOE4* carriers [84]. When the waist-to-hip ratio (WHR) was used as a measure of obesity, *APOE4* and a higher WHR resulted in a significantly worse executive and memory functions [85]. *APOE4* carriers are also less responsive to AD insulin therapies. When AD and MCI patients were given intranasal insulin, non-*APOE4* carriers exhibited improvements in verbal memory while *APOE4* carriers exhibited a decline in verbal memory [86]. Memory impaired non-*APOE4* carriers also had an increase in plasma Aβ42 levels at each dose, which directly correlated with an increased insulin dose, while *APOE4* carriers only had increased plasma Aβ42 after the lowest dose [86]. Intravenous insulin treatment for AD patients had similar results with non-*APOE4* carriers having increased memory facilitation and decreased APP levels, while *APOE4* carriers showed no cognitive differences, and increased APP levels [87]. Thus, human studies show *APOE4* carriers are more affected by obesity through metabolic alterations, cognition, and AD pathology.

Several studies have examined the metabolic and cognitive alterations associated with the *APOE* genotypes and weight gain in rodent models, testing conclusions from human studies. Both *APOE* knock in (*APOE* KI) and *APOE* knock out (*APOE* KO) mice have been useful in studying the effects of obesity and HFD. Compared to *APOE3-ob/ob* mice, male *APOE4-ob/ob* mice exhibited increased plasma insulin and insulin resistance similar to *APOE4* mice with diet induced obesity [88]. *APOE4-ob/ob* mice also had increased fatty liver and hepatic triglycerides compared to *APOE3-ob/ob* mice [88]. Both *APOE3-ob/ob* and *APOE4-ob/ob* mice experienced significant weight gain, with *APOE3-ob/ob* mice gaining more [88]. Female *APOE3* KI mice fed a high fat western diet showed weight gain, increases in fasting cholesterol, insulin, and glucose levels, and decreases in glucose clearance and responsiveness to insulin as compared to C57BL/6 on HFDs [89]. *APOE* KO mice have been integral in understanding the function of APOE in metabolism. *APOE* KO mice have decreased lipoprotein clearance, increased plasma cholesterol [90], and decreased adipose triglyceride levels [91]. They also have an increase in inflammation, extracellular matrix degradation and rapidly developing atherosclerosis [90]. *APOE* KO mice on HFD had increases in plasma cholesterol, but, unlike the *APOE3* mice, there was no alteration in glucose clearance or response to insulin, and only slightly elevated fasting glucose levels [89]. This study shows that female *APOE3* mice respond differentially to a HFD when compared to control mice or mice lacking *APOE*. Another study of male *APOE3* and *APOE4* KI mice on a high fat western diet [75] showed weight gains, with a stronger effect in *APOE3* mice, but no differences in baseline glucose and insulin levels by *APOE* genotype [75]. However, when tested for glucose tolerance, *APOE4* mice on the HFD had more difficulty metabolizing the excess glucose and had a decreased response to insulin when compared to the *APOE3* mice [75]. These data indicate a more robust metabolic alteration in the *APOE4* mice, consistent with the pattern in humans with *APOE3* mice weighing more than *APOE4* mice regardless of diet [92].

The previous study did not directly compare the *APOE3* and *APOE4* mice on the HFD to the *APOE3* and *APOE4* mice on low fat ingredient matched chow, but a study by Johnson et al. made this comparison [93]. Metabolically, both *APOE3* and *APOE4* female mice on the HFD gained a significant amount of weight with *APOE3* mice gaining more [93], in agreement with the previous study [75]. These mice had increased fasting blood glucose and insulin levels, with an exaggerated glucose intolerance in the *APOE4* mice [93]. The HFD mice also exhibited cognitive deficits with decreased time spent with the novel object, and decreased latencies to escape on the Morris Water Maze, indicating deficits in both spatial navigation and recognition learning [93]. *APOE4* mice on the HFD once again had exaggerated spatial deficits; however, these deficits were reversed with a low fat dietary intervention [93]. Similarly, female *APOE3* and *APOE4* mice on a HFD experienced increased insulin resistance and glucose intolerance [94]. *APOE4* mice on the HFD experienced lower cerebral blood volume and increased deficits in the Morris Water Maze when compared to *APOE3* mice on a HFD [94].

Similarly, Pike et al. examined the effects of a HFD on male *APOE3* and *APOE4* on the 5xFAD (EFAD) background [95]. There was a decrease in glucose clearance in both the E3FAD and E4FAD mice on the HFD [95]. The E4FAD mice had more amyloid deposits when compared to E3FAD mice, and the E4FAD mice on the HFD had increased amyloid when compared to both the mice on the standard diet and E3FAD mice on the HFD [95]. Consistent with higher amyloid levels, the E4FAD mice had more microglia in the hippocampus and entorhinal cortex when compared to the mice on a standard diet and *APOE3* mice on the HFD [95]. This study indicates that the effects of HFD on AD pathology is stronger in the presence of *APOE4*.

Finally, another study examined the cognitive and neuroinflammatory difference between C57BL/6, *APOE4* and *APOE*^−/−^ mice on a HFD. Female mice on a HFD for three months were tested on the Morris Water Maze. When on the HFD, the WT mice had better performance in the task when compared to the *APOE*^−/−^ mice, with a decreased latency to escape [96]. Surprisingly, the *APOE4* mice on the HFD did not exhibit signs of hindered memory acquisition or retention [96]. When examining the mice for inflammation, *APOE4* mice on a standard diet had higher levels of hippocampal CD68, which was decreased with a HFD [96]. Overall, this study suggests that a HFD does not adversely affect *APOE4* mice in terms of cognition and inflammation. This finding is unexpected considering the increased metabolic alterations experienced by the *APOE4* mice on HFDs, and the other studies displaying cognitive deficits in *APOE4* mice [93,94].

Thus, although there are contrasting findings, overall studies in mouse models mimic the results of the human studies, with the *APOE4* carriers being at a higher risk of metabolic effects of HFD. These consistencies and differences emphasize the relevance of this animal model and the need to study this interaction. 

## 5. Conclusions

**Figure 1:**
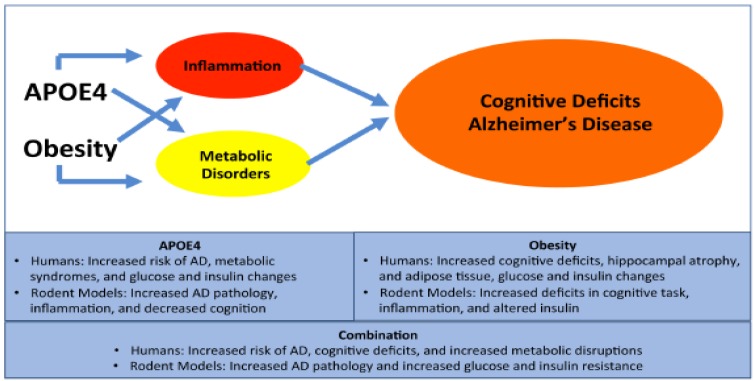
Contribution of Apolipoprotein E (*APOE*)4 and obesity to Alzheimer’s Disease (AD). APOE4 and obesity independently contribute to inflammation and metabolic disorders. The inflammation and metabolic disorders can then lead to increased cognitive deficits and AD. Human and rodent studies both support these claims, and that APOE4 and obesity synergistically increase cognitive deficits and AD risk.

*APOE4* genotype and obesity have both been linked to an increased risk to developing AD. Both human and rodent models have repeatedly shown increases in cognitive deficits, inflammation and sex dependent differences associated with *APOE4* and obesity independently. These findings are summarized in Figure 1. However, the interactions between obesity and *APOE* genotypes are less clear. Multiple studies have examined the metabolic and cognitive effects of *APOE* genotypes and obesity, but there are few studies linking the effects of HFD on both metabolism and cognition under the same conditions in terms of age and sex. Furthermore, the question of the mechanisms behind the effects of diet on cognition remains. One potential mechanism could be peripheral metabolic alterations leading to altered brain glucose utilization [97,98]. Changes in the peripheral glucose and insulin tolerance can affect the tolerance in the brain, and the deficits already exhibited in *APOE4* carriers may be further exacerbated with the strain of a HFD. Another possible mechanism is related to inflammation from peripheral adipose tissue. Peripheral adipose tissue releases multiple inflammatory cytokines such as TNF-α and IL-6 [5,31], which could act as a chronic systemic injury, resulting in increased BBB permeability [99]. More comprehensive studies are needed to examine both metabolic alterations and cognition in obese individuals of different *APOE* genotypes. These studies would allow clarification of the inconsistent findings above, and would test whether the observations above are due to interactions between metabolic syndrome and cognition across *APOE* genotypes.
